# Prospective evaluation of functional outcomes in 395 patients with an ileal neobladder 1 year after radical cystectomy

**DOI:** 10.1007/s00345-023-04520-x

**Published:** 2023-07-15

**Authors:** Henning Bahlburg, Fabian Schuster, Karl Heinrich Tully, Marius Cristian Butea-Bocu, Moritz Reike, Florian Roghmann, Joachim Noldus, Guido Mueller

**Affiliations:** 1 Department of Urology, Marien Hospital Herne, Ruhr-University Bochum, Hölkeskampring 40, 44625 Herne, Germany; 2 Center for Urological Rehabilitation, Kliniken Hartenstein, Bad Wildungen, Germany

**Keywords:** Urinary incontinence, Erectile dysfunction, Functional outcome, Inpatient rehabilitation, Ileal neobladder

## Abstract

**Purpose:**

This study aims to report on functional outcomes in a large cohort of patients who underwent inpatient rehabilitation (IR) in a highly specialized, high-volume German urologic rehabilitation center after radical cystectomy (RC) and creation of an ileal neobladder (INB).

**Methods:**

Data for 842 patients, who underwent three weeks of IR after RC and urinary diversion between April 2018 and December 2019 were prospectively collected. INB patients were surveyed on continence and sexual function. Data were collected at 4 weeks (T1), 6 months (T2), and 12 months (T3) after RC. Multivariate logistic regressions were performed to identify predictors of better functional outcomes.

**Results:**

INB was chosen as urinary diversion in 395 patients (357 male, 38 female). Social continence (maximum of one safety pad/24 h) was reported by 78.3% of men and 64.0% of women at T3. Severe incontinence was reported by 27.3% of men and 44.0% of women. Male sex was identified as an independent predictor for the use of no pads at T3 (OR 4.110; 95% CI 1.153–14.655; p = 0.029). Nerve-sparing surgery was identified as an independent predictor both for the use of only a safety pad (OR 1.918; 95% CI 1.031–3.569; p = 0.040) and good erectile function at T3 (OR 4.377; 95% CI 1.582–12.110; p = 0.004).

**Conclusion:**

Urologists should aspire for nerve-sparing surgery. When advising patients before RC, functional outcomes (continence, sexual function) should be given special attention. Women should be counseled on potentially prolonged urinary incontinence.

## Introduction

It is known that in patients with an ileal neobladder (INB), urinary continence is an independent prognostic factor for a high quality of life (QoL) post-surgery, while incontinence may lead to diminished body image [1, 2]. In current literature, continence rates after creation of an INB vary from 49 to 96% during the daytime and 35–95% at night. Continence rates increase over time and are higher in men than in women [2, 3]. An impaired emotional and cognitive function in patients with an INB due to urinary incontinence has been reported [2]. A low-pressure reservoir and an intact external urethral sphincter are important factors for postoperative continence [4]. Continence in general, but especially during daytime, is improved by nerve-sparing surgery [5–7]. Several factors such as water diffusion from the intestinal wall into the reservoir, a decreased sphincter tonus, and peaks in intraluminal pressure by increased peristaltic movements have been discussed as influencing nocturnal incontinence [8–10]. As function of the external urethral sphincter deteriorates with age, patients younger than 65 reach higher continence rates [11–13]. Accordingly, the study by Erdogan et al. identified increasing age, diabetes, and a body mass index ≥ 30 kg/m^2^ as independent negative predictors of urinary continence in the early postoperative period [7].

When performing radical cystectomy (RC), nerve-sparing surgery should be aspired for when feasible as stated in the current guideline by the European Association of Urology [14]. In addition to urinary incontinence, erectile dysfunction (ED) following surgery may also negatively influence quality of life [5, 15, 16].

So that patients can reach the important goal of reintegration into daily life, German social laws entitle cancer patients to an average of 3 weeks of inpatient rehabilitation (IR). The guideline of the German Society of Urology recommends that all patients be offered several weeks of IR after RC for bladder cancer to minimize functional disorders [17]. It is assumed that in Germany almost all patients participate in IR as recommended. The design of our study allows us to report on the functional outcomes of a large number of INB patients in a recent period.

## Methods

This prospective study was based on clinical data of patients with urothelial carcinoma of the bladder who had received RC and INB creation in various hospitals across Germany and who were treated in a specialized center for urological rehabilitation (Kliniken Hartenstein, Bad Wildungen, Germany) between April 2018 and December 2019. The study protocol was approved by an institutional research committee (research authorization number FF30/2017). At the beginning of IR (T1) and at both 6 (T2) and 12 months (T3) after surgery, continence and erectile function were measured by validated questionnaires.

### Inpatient rehabilitation (IR)

During IR, patients were treated by specialized physiotherapists regarding urinary continence. The multimodal continence therapy includes osteopathic physiotherapy, external urethral sphincter exercises, and educational measures on neobladder management and care (e.g. micturition diary with instruction to empty the neobladder initially every 2–3 h during the day as well as at night; careful increase of neobladder volume to achieve sensitivity concerning neobladder volume; and prevention of residual urine volume with special mechanisms for emptying the neobladder). For patients without improvement in daytime continence within two weeks of therapy, video-assisted biofeedback-sphincter training via transurethral endoscopy can be performed [7].

### International Consultation on Incontinence Questionnaire-Short Form (ICIQ-SF)

The ICIQ-SF is a validated patient-reported assessment and examines the frequency and quantity of involuntary loss of urine and its influence on QoL [18]. The questionnaire consists of three scored items, scored on a Likert-scale between 0 and 5. Zero points are equivalent to no impairment while a score of 5 points is equivalent to a very high impairment. The total sum of all three scored items allows for classifying the patients’ incontinence into three groups. A sum between 1 and 5 points is defined as mild, a score between 6 and 10 points as moderate, and a score ≥ 11 as severe incontinence [19]. Additionally, the number of pads used daily is examined. Social continence was defined as the use of a maximum of one safety pad per 24 h.

### International Index of Erectile Function (IIEF-5)

The IIEF-5 is a validated, patient-reported tool to measure erectile function and was developed based on the International Index of Erectile Function, containing 15 items [20]. Its five items concern erectile function, sexual desire, orgasm ability, and sexual satisfaction. Each question is scored on a Likert-scale of 1–5. 1 point equals a high impairment, and 5 points equals no impairment. The erectile function can then be classified according to the sum of all scores. A score of 5–7 points signals severe, a score between 8 and 11 moderate, a score of 12–16 weak to moderate, a score of 17–21 weak, and a score of 22–25 points no erectile dysfunction (ED).

### Erection Hardness Score (EHS)

The EHS offers another easy tool to measure erectile function, in which the erection is scored on a 5-point Likert-scale (0–4) [21]. The result can be interpreted as follows. Zero points: the penis does not enlarge when the patient is sexually aroused. One point: the penis enlarges but does not get hard. Two points: the penis is hard but not sufficiently so for penetration. Three points: the penis is hard enough for penetration but not totally hard. Four points: the penis is hard and rigid.

### Statistical analyses

The Wilcoxon test and a Chi-squared test (McNemar) were used to assess the level of significance regarding changes during follow-up in quantitative variables and proportions. Multivariate logistic regression was performed to identify predictors for “safety pad” and “no pad” 1 year after surgery. Significance was considered at p < 0.05. Analyses were performed using SPSS, version 29 (IBM, Chicago, IL, USA).

## Results

A total of 842 patients after RC from 135 different hospitals in Germany underwent IR in the aforementioned timeframe, of which 395 patients (46.9%) received an INB. Their characteristics are summarized in Table 1. The median age was 64 years (IQR 58–69). The majority of patients were male (90.4%). IR started at a median of 28 days (IQR 23–35) and ended with a median of 54 days (IQR 48–62) after surgery. The response rates for the follow-up survey were 83.0% (n = 328) at T2 and 71.6% (n = 283) at T3.

**Table 1:** Characteristics of 395 patients after radical cystectomy and creation of an ileal neobladder

Variable	Value
Patients, n (%)	395 (100)
Age (years), Median (IQR)	64 (58–69)
≤ 59 years, n (%)	123 (31.1)
60–69 years, n (%)	182 (46.1)
≥ 70 years, n (%)	90 (22.8)
Sex, n (%)	
Male	357 (90.4)
Female	38 (9.6)
BMI (kg/m^2^), Median (IQR)	25 (23–27)
< 30, n (%)	353 (89.4)
≥ 30, n (%)	42 (11.6)
Cardiovascular disease, n (%)	221 (55.9)
Diabetes, n (%)	42 (10.6)
Neoadjuvant chemotherapy, n (%)	46 (11.6)
Method of surgery, n (%)	
Open cystectomy	348 (88.1)
Robot-assisted cystectomy	47 (11.9)
Tumor stage, n (%)	
≤ pT2	300 (75.9)
≥ pT3	95 (24.1)
Lymph node positive, n (%)^a^	45 (11.8)
No. of lymph nodes removed, Median (IQR)	17 (12–24)

*IQR* interquartile range, *BMI* body mass index

^a^Data available for 382 patients

### Continence

Data on continence outcomes for men enrolled in this study can be found in Table 2a.

**Table 2:** Continence after radical cystectomy and creation of an ileal neobladder

	T1	T2	T3
(a) Male patients			
ICIQ Score, Median (IQR)	15 (12–18)	7 (4–11)*	7 (4–11)
Continence (score 0), n (%)	5 (1.5)	34 (11.5)*	36 (14.2)
Mild incontinence (score 1–5), n (%)	14 (4.1)	87 (29.5)*	72 (28.5)
Moderate incontinence (score 6–10), n (%)	46 (13.4)	91 (30.8)*	76 (30.0)
Severe incontinence (score ≥ 11), n (%)	279 (81.1)	83 (28.1)*	69 (27.3)
Pads at day, Median (IQR)	4 (2–6)	1 (1–2)*	1 (1–2)
Pads at night, Median (IQR)	3 (2–4)	1 (1–2)*	1 (1–2)*
“Safety pad”, n (%)	68 (19.0)	135 (45.8)*	109 (42.9)
“No pad”, n (%)	5 (1.5)	93 (31.5)*	90 (35.4)
(b) Female patients			
ICIQ Score, Median (IQR)	10 (10–18)	13 (5–19)	8 (3–13)
Continence (score 0), n (%)	4 (10.5)	3 (10.3)	5 (20)
Mild incontinence (score 1–5), n (%)	3 (7.9)	5 (17.2)	6 (24.0)
Moderate incontinence (score 6–10), n (%)	3 (7.9)	5 (17.2)	3 (12.0)
Severe incontinence (score ≥ 11), n (%)	28 (73.7)	16 (55.2)*	11 (44.0)
Pads at day, Median (IQR)	5 (4–8)	4 (2–6)*	2.5 (1–5)
Pads at night, Median (IQR)	3 (2–4)	2 (1–4)*	1 (1–2.5)*
“Safety pad”, n (%)	6 (15.8)	9 (31.0)	13 (52.0)
“No pad”, n (%)	2 (5.3)	3 (10.3)	3 (12.0)

Significant results are shown in bold

(Wilcoxon-test or Chi squared test (McNemar) as appropriate)

*ICIQ* International Consultation on Incontinence Questionnaire (short form), *T1* 4 weeks after surgery (data available for 344 men and 38 women), *T2* 6 months after surgery (data available for 295 men and 29 women), *T3* 12 months after surgery (data available for 253 men and 25 women)

^*^p < 0.05 for the difference to the last time of evaluation

At T1, men reached a median score of 15 (IQR 12–18) in the ICIQ-SF. Continence improved significantly to a median score of 7 (IQR 4–11, p < 0.05) 6 months post-surgery. 12 months post-surgery, a median score of 7 (IQR 6–10) was reported, signaling moderate incontinence. At T1, 1.5% (n = 5) reported complete continence. A significant increase in men with complete continence (ICIQ-score = 0) was seen at T2 (11.5%). Light incontinence (ICIQ = 1–5) was reported by 29.5% of men. Accordingly, the rate of men with severe incontinence (ICIQ =  ≥ 11) decreased from 81.1% at T1 to 28.1% at T2. During further follow-up, no statistically significant improvements were detected. 14.2% of men were continent 12 months post-surgery, while 28.5% reported light incontinence. Thirty percent reported moderate incontinence, and 27.3% had severe incontinence. At T1, men used a median of 4 pads during the day (IQR 2–6) and 3 pads (IQR 2–4) during the night. Use of pads decreased significantly to 1 pad during the day and 1 pad during the night at T2 (IQR 1–2; p < 0.05). The number of pads used remained the same at T3. The percentage of men using a safety pad increased significantly to 45.8% at T2 (p < 0.05). At T2, a significantly higher portion of men did not need any safety pad (31.5%, p < 0.05). Social continence was reported by 78.3% of men at T3.

Data on continence rates in female participants can be found in Table 2b. Female patients reached a median ICIQ score of 8 points (IQR 3–13) 12 months post-surgery. Twenty percent of women reported complete continence at T3. However, 44.0% were still limited by severe incontinence (ICIQ ≥ 11) at this point. Median use of pads decreased to 2.5 (IQR 1–5) during the day and 1 pad (IQR 1–2.5) during the night. At T3, 52.0% of women used only a safety pad, while 12.0% used no pad at all. Thus, social continence was reached by 64.0% of women included in this study.

Multivariate regression analysis (Table 3) identified nerve-sparing surgery as an independent positive predictor for the use of a safety pad only at T3 (OR 1.918; 95% CI 1.031–3.569; p = 0.040). Male sex was identified as an independent positive predictor for the use of no pads at T3 (OR 4.110; 95% CI 1.153–14.655; p = 0.029).

**Table 3:** Multivariate regression analysis to identify independent predictors for “safety pad” and “no pad” 1 year after radical cystectomy and creation of an ileal neobladder

Variable	“Safety pad”		“No pad”	
	OR (95% CI)	p	OR (95% CI)	p
Age ≤ 59 years vs. ≥ 60 years	0.967 (0.515–1.816)	0.917	1.359 (0.752–2.454)	0.310
Male vs. female	0.719 (0.301–1.717)	0.458	4.110 (1.153–14.655)	**0.029**
BMI ≥ 30 kg/m^2^ (yes vs. no)	0.601 (0.235–1.538)	0.288	1.194 (0.481–2.962)	0.703
Diabetes (yes vs. no)	0.609 (0.222–1.669)	0.335	1.575 (0.624–3.978)	0.336
Cardiovascular disease (yes vs. no)	1.709 (0.965–3.026)	0.066	0.605 (0.343–1.065)	0.081
Open vs. Robot-assisted surgery	1.045 (0.479–2.284)	0.911	1.845 (0.782–4.353)	0.162
Nerve sparing (yes vs. no)	1.918 (1.031–3.569)	**0.040**	0.967 (0.544–1.719)	0.909

Significant results (p < 0.05) are shown in bold

*OR* odds ratio, *CI* confidence interval, *BMI* body mass index

### Erectile function

Information on erectile function pre-surgery was available for 99.2% of men. Before surgery, 77.4% of men reported an erectile function sufficient for sexual intercourse (EHS ≥ 3). Data on erectile function are shown in Table 4.

**Table 4:** Erectile function after radical cystectomy and creation of an ileal neobladder evaluated by IIEF-5 and EHS

All patients	T1	T2	T3	Patients with EHS ≥ 3 before surgery and NS	T1	T2	T3
IIEF-5 score, available for	n = 271	n = 254	n = 217	IIEF-5 score, available for	n = 82	n = 94	n = 83
Median (IQR)	6 (5–7)	5 (5–7)	5 (5–8)	Median (IQR)	7 (6–9)	6 (5–10)	7 (5–13)
22–25, n (%)	12 (4.4)	7 (2.8)	7 (3.2)	22–25, n (%)	9 (11.0)	6 (6.4)	6 (7.2)
17–21, n (%)	13 (4.8)	8 (3.1)	11 (5.1)	17–21, n (%)	4 (4.9)	5 (5.3)	7 (8.4)
12–16, n (%)	10 (3.7)	15 (5.9)	17 (7.8)	12–16, n (%)	**2 (2.4)**	10 (10.6)	14 (16.9)^*^
8–11, n (%)	29 (10.7)	33 (13.0)	26 (12.0)	8–11, n (%)	11 (13.4)	15 (16.0)	11 (13.3)
5–7, n (%)	207 (76.4)	191 (75.2)	156 (71.9)	5–7, n (%)	56 (68.3)	58 (61.7)	45 (54.2)
EHS, available for	n = 272	n = 270	n = 220	EHS, available for	n = 84	n = 99	n = 83
EHS ≥ 3, n (%)	15 (5.5)	25 (9.3)	24 (10.9)	EHS ≥ 3, n (%)	10 (11.9)	17 (17.2)	16 (19.3)

Significant results are shown in bold

*IIEF-5* International Index of Erectile Function (5-item version), *EHS* erection hardness score (≥ 3: erectile function sufficient for sexual intercourse), *NS* nerve sparing, *T1* 4 weeks after surgery, *T2* 6 months after surgery, *T3* 12 months after surgery

^*^p = 0.002 for T1 vs. T3 calculated by the Chi-squared test (McNemar)

### IIEF-5

Results showed that more than 70% of men were suffering from severe ED during follow-up. Only 8.3% reported no or only a weak ED (IIEF-score ≥ 17).

Before surgery, 116 men (median age 60 years, IQR 55–66) had a sufficient erection (EHS ≥ 3) and underwent nerve-sparing surgery. Weak to moderate ED was reported by 2.4% of these men at T1. However, this portion increased significantly to 16.9% at T3. At T3, only 15.6% reported no or only a weak ED, while 54.2% suffered from complete ED. Multivariate regression analysis (Table 5) identified nerve-sparing surgery as the only independent predictor for erectile function 1 year after RC (OR 4.377; 95% CI 1.582–12.110; p = 0.004). A trend towards significance was observed when comparing robot-assisted and open RC (p = 0.051).

**Table 5:** Multivariate regression analysis to identify independent predictors for an erectile function fit for sexual intercourse (EHS ≥ 3) 1 year after RC

Variable	OR (95% CI)	p
Age ≤ 59 years vs. ≥ 60 years	1.179 (0.448–3.105)	0.739
BMI ≥ 30 kg/m^2^ (yes vs. no)	3.718 (0.818–16.892)	0.089
Diabetes (yes vs. no)	0.961 (0.188–4.924)	0.962
Cardiovascular disease (yes vs. no)	1.321 (0.484–3.606)	0.587
Robot-assisted vs. open surgery	2.835 (0.994–8.087)	0.051
Nerve sparing (yes vs. no)	4.377 (1.582–12.110)	**0.004**

Significant results (p < 0.05) are shown in bold

*EHS* erection hardness score, *OR* odds ratio, *CI* confidence interval, *BMI* body mass index

### EHS

4 weeks after surgery, 5.5% of men reported an erectile function sufficient for sexual intercourse (EHS ≥ 3). During follow-up, this cohort steadily grew. At T3, 10.9% reported an EHS ≥ 3. In men who had a pre-surgery EHS ≥ 3 and underwent nerve-sparing surgery, 19.3% reported an EHS ≥ 3 at T3.

### Comparison of functional outcomes in relation to nerve-sparing surgery (Table 6)

**Table 6:** Functional outcomes in men 1 year after surgery related to  nerve-sparing surgery

Variable	NS	nNS	p^a^
Age (years), median (IQR)	60.5 (55.5–67.0)	65.0 (60.0–70.0)	** < 0.001**
ICIQ-Score	6.5 (4.0–11.0)	7.0 (4.0–11.0)	0.291
“Social continence” (≤ 1 pad in 24 h)	57 (58.2)	111 (69.8)	0.057
“No pad”, n (%)	36 (36.7)	54 (34.8)	0.759
“Safety pad”, n (%)	21 (21.4)	57 (35.8)	**0.015**
EHS ≥ 3, n (%)	17 (18.5)	7 (5.4)	**0.002**

Significant results (p < 0.05) are shown in bold

Data concerning “social continence” available for 159 men with nNS and 98 men with NS

Data concerning “no pad” available for 155 men with nNS and 98 men with NS

Data concerning “safety pad” available for 159 men with nNS and 98 men with NS

Data concerning EHS available for 130 men with nNS and 92 men with NS

*NS* nerve-sparing surgery, *nNS* non-nerve-sparing surgery, *EHS* erection hardness score (≥ 3: erectile function fit for sexual intercourse)

^a^Mann–Whitney U test or Chi-squared test (Pearson) as appropriate

When comparing functional results of men undergoing nerve-sparing surgery to those of men without nerve-sparing surgery, several significant differences were detected. Firstly, men undergoing nerve-sparing surgery were significantly younger (median age 60.5 years (IQR 55.5–67) vs. 65 years (IQR 60–70); p < 0.001). Furthermore, after nerve-sparing surgery, patients were significantly less likely to need a safety pad (21.4% vs 37%; p = 0.008) and more likely to report an erectile function sufficient for sexual intercourse (EHS ≥ 3; 18.5% vs 5.4%; p = 0.002).

## Discussion

After RC and creation of an INB, urinary continence may be the most important factor for a high QoL [2]. According to our data, social continence (0–1 pad per 24 h) was reached by 78.3% of men and 64.0% of women 12 months after creation of an INB. The difference in continence rates between genders is backed up by data from the literature [2, 3]. Multivariate regression analysis identified nerve-sparing surgery as an independent predictor for the use of one safety pad 12 months post-surgery, while male sex was identified as an independent predictor for the use of no pads. Clifford et al. reported on 188 patients, of which 49% reached social continence 18–36 months post-surgery [12]. Novara et al. reported on 113 patients, who had surgery between 2002 and 2006 and were evaluated by the ICIQ. In this cohort, 18% reached complete continence, while 68% did not use any pads [22]. Ahmadi et al. examined 179 men with an ileum neobladder 12 months post-surgery. In this cohort, 15% did not use any pads [23]. The larger cohort in our study and improved surgical technique (laparoscopic, robot-assisted) with high-definition, 3D vision, may explain a higher rate of socially continent patients than in the aforementioned studies. Furthermore, structured urologic rehabilitation in a specialized center may also influence functional outcomes after RC.

Data on sexual function after radical cystectomy vary enormously. Schoenberg et al. reported an improved sexual function in younger men over time with potency rates of up to 50% 10 years after surgery [24]. According to current literature, potency rates up to 65% can be reached in carefully selected collectives [6]. However, other studies showed potency rates of 10–20% only [22, 25]. Even if nerve-sparing surgery is not possible, up to 35% of men may still have sufficient erectile function [26]. Nonetheless, according to an American study, only 15% of men receive sufficient treatment for their ED [27].

Before surgery, 77.4% of men in the present cohort were potent (EHS ≥ 3). However, only 19.3% of men who were potent before surgery and underwent nerve-sparing surgery had sufficient erectile function 12 months post-surgery. At the same time, only 8.3% of all patients suffered from no or weak ED (IIEF-5 score ≥ 17). On the contrary, about 70% of all men reported a severe ED (IIEF-5 score 5–7) during follow-up. As identified by multivariate regression analysis, nerve-sparing surgery is the only independent predictor for good erectile function 12 months after surgery. After nerve-sparing surgery, a significantly greater portion of men reported good erectile function, and they were less likely to use a safety pad. About 50% of men aged 65–75 are still sexually active [28]. There is a desire among cancer patients to gain information about the influence of cancer and its respective treatment on their sexuality. However, patients often refrain from talking about their sexual desires and medical professionals seldom raise this issue [29]. If applied, a structured penile rehabilitation including andrological examinations, intake of phosphodiesterase inhibitors, use of vacuum pumps, and intracavernous injections with Alprostadil may lead to improved sexual function 12 months after surgery [30].

In this study, patients from various urologic departments with different levels of experience from all over Germany were included. An improved functional outcome may be achieved when surgery is performed in specialized centers. However, this was not evaluated in this study. Information about nerve-sparing surgery was taken from discharge papers and surgical reports and could thus not be independently validated. The actual percentage of patients who had nerve-sparing surgery may be lower than reported, leading to worse results concerning erectile function.

Nonetheless, by using validated questionnaires, this study offers a unique insight into continence and potency rates 4 weeks, 6 months, and 12 months after RC and creation of an INB. Currently, about 1600 patients undergo RC with creation of an INB in Germany annually. Almost one quarter of these patients is included in this study. Thus, our study reports real-world data acquired in a recent period. Even if nerve-sparing surgery is performed, potency rates are low. Continence rates improve over time. However, more than 20% of male patients and more than 35% of female patients with an INB still report relevant urinary incontinence 12 months after surgery.

## Conclusion

When advising patients concerning urinary diversion, functional outcomes concerning continence and sexual function should receive special attention. In particular, female patients should be informed about potentially prolonged incontinence.

## Data Availability

Data are not publicly available but can be made available by the corresponding author upon reasonable request.
